# Addition of progesterone to feminizing gender-affirming hormone therapy in transgender individuals for breast development: a randomized controlled trial

**DOI:** 10.1186/s40360-023-00724-4

**Published:** 2023-12-20

**Authors:** Benthe A.M. Dijkman, Danithsia Helder, Lidewij S. Boogers, Noor C. Gieles, Jason O. van Heesewijk, Sjoerd te Slaa, Niels P.T.J. Liberton, Chantal M. Wiepjes, Christel J.M. de Blok, Martin den Heijer, Koen M.A. Dreijerink

**Affiliations:** 1 Department of Endocrinology and Metabolism, Center of Expertise on Gender Dysphoria, Endo-ERN Reference Center; Amsterdam UMC, location VU University, De Boelelaan 1117, 1081 HV Amsterdam, The Netherlands; 2 Research Institute Amsterdam Gastroenterology, Endocrinology and Metabolism, Amsterdam UMC, location VU University, De Boelelaan 1117, 1081 HV Amsterdam, The Netherlands; 3 Department of Medical Technology, 3D Innovation Lab, Amsterdam UMC, location VU University, Van der Boechorststraat 7, 1081 BT Amsterdam, The Netherlands

**Keywords:** Transgender, Gender-affirming hormone therapy, Breast development, Breast volume, Progesterone, Estradiol

## Abstract

**Background:**

Feminizing gender-affirming hormone therapy (GAHT) for transgender individuals traditionally includes estradiol and androgen deprivation. Research has demonstrated that breast size as a result of GAHT in transgender women is often limited. Therefore, transgender women often choose to undergo breast augmentation surgery. Progesterone is important for breast development in cisgender women during puberty. A potential role for progesterone in breast development in transgender women has not been investigated in a randomized controlled experimental set-up. The primary objective of this study is to explore the effects on breast volume of addition of oral progesterone to GAHT with estradiol in transgender women after vaginoplasty or orchiectomy. Secondary objectives include assessment of safety, satisfaction, mood, sleep and sexual pleasure.

**Methods:**

This is a non-blinded, non-placebo, randomized controlled trial using a factorial design in adult transgender individuals assigned male sex at birth who have undergone GAHT for at least one year and underwent vaginoplasty or orchiectomy. The study design allows for rapid assessment of potential synergistic effects of various dose combinations of estradiol and progesterone on breast volume change: Ninety participants will be randomized into six groups of 15 subjects each, receiving either the baseline dose of estradiol, the baseline dose of estradiol and progesterone 200 mg daily, the baseline dose of estradiol and progesterone 400 mg daily, twice the baseline dose of estradiol, twice the baseline dose of estradiol and progesterone 200 mg daily or twice the baseline dose of estradiol and progesterone 400 mg daily, all for a duration of 12 months. The main study parameters include changes in breast volume as determined by 3D measurements. Participants will be followed-up with laboratory testing including serum progesterone concentrations as well as surveys for satisfaction, mood, sleep quality and sexual pleasure.

**Discussion:**

This study will indicate whether progesterone is safe and of additional value with regard to breast volume change in transgender individuals receiving feminizing GAHT. The results of this study will be useful for innovation of feminizing GAHT.

**Trial registration:**

WHO International Clinical Trials Registry Platform: EUCTR2020-001952-16-NL; date of registration: 12 December 2020 https://trialsearch.who.int/Trial2.aspx?TrialID=EUCTR2020-001952-16-NL.

## Background

Transgender people experience an incongruence between their sex assigned at birth and their gender identity. In the Netherlands, it is estimated that 1:2,800 birth-assigned males and 1:5,200 birth-assigned females undergo or have undergone gender-affirming treatment [1]. Transgender individuals with the male sex assigned at birth who receive feminizing gender-affirming hormone therapy (GAHT) traditionally use estradiol and anti-androgenic treatment before they can choose to undergo vaginoplasty or orchiectomy [2, 3]. In this report, we will use the term transgender women for individuals who were assigned the male sex at birth and use feminizing hormone therapy. After gender-affirming surgery, hormone therapy replaces endogenous hormone production. Research has demonstrated that breast size as a result of GAHT in transgender women is often limited and that therefore transgender women often choose to undergo breast augmentation surgery [4, 5].

Progesterone is the natural ligand of the progesterone receptor (PR). In both cisgender (alignment of sex as-signed at birth and gender identity) men and women progesterone is synthesized in the adrenal glands and in pre-menopausal cisgender women the primary source is in the ovaries. The estrogen receptors and progesterone receptor (PR) are expressed in many tissues in the human body and regulate gene expression and cell differentiation. In addition to estradiol, progesterone has an important role in mammary and breast development during female puberty: Estradiol stimulates the expansion of breast tissue and progesterone stimulates differentiation of the mammary tissue [6, 7]. A potential role for progesterone has not been investigated with regard to breast development in transgender women in a randomized controlled fashion. In addition to estradiol, transgender women are frequently treated with anti-androgens. In our center, in the past, cyproterone acetate, an anti-androgen with progestogenic characteristics - now replaced with GnRH agonists - was most often used to lower testosterone levels before vaginoplasty or orchiectomy, which is a reason progesterone has not been included in the treatment. In other countries, in particular in the United States where cyproterone acetate is not available, progesterone is more often added to GAHT. However, whether this has a beneficial effect on breast development is not clear as evidence is lacking [8]. Since the addition of progesterone is theoretically beneficial, this is a persisting subject of international debate.

Only few studies have addressed the effect of addition of the progestin medroxyprogesterone acetate (MPA) to estradiol (and anti-androgen) treatment in transgender women: In a small group of transgender women, no differences in breast size were observed relative to transgender women receiving estrogens only [9]. In a retrospective uncontrolled study, transgender women reported increased breast volume when using MPA. Side effects were rare and most frequently included mood swings [10]. In 2019, Prior suggested oral natural progesterone to have several additional feminizing effects when incorporated into the hormonal treatment of transgender women, including increased breast and areolar size. Furthermore, progesterone might have beneficial effects regarding cardiovascular and breast cancer risk, mood, and sleep quality [11]. In cisgender women, the progesterone surge during the luteal phase is associated with a decrease of sexual desire [12]. In a recent retrospective study, progesterone was not shown to make any discernable difference in satisfaction with libido compared with standard GAHT regimens [13]. Also, a case-control study was published in which participants received a low dose of 100 mg micronized progesterone in addition to estradiol GAHT for a period of three months. In this study, no significant breast volume changes were observed [14].With regard to side effects, progestin use has been linked to decreased high-density lipoprotein (HDL) cholesterol levels in transgender women [15]. In general, transgender women have a lower risk of developing breast cancer, compared with cisgender women [16]. The hormone progesterone is widely used as hormone replacement therapy in cisgender women and during in vitro fertilization procedures and has not been demonstrated to increase breast cancer risk [17]. The use of micronized progesterone has not been associated with increased risk of venous thromboembolism in cisgender women [18].

This study aims to explore the effects of addition of progesterone to estradiol treatment and increasing estradiol doses on breast volume with regard to breast size in transgender women. Secondary objectives include assessment of the effects of progesterone combined with estradiol on satisfaction with breast volume, mood and sleep quality, sexual pleasure, side effects of the treatment regimen, as well as serum progesterone concentrations.

## Methods/design

### Aim, design and setting of the study

In order to determine the effects and side effects of adding oral progesterone to feminizing GAHT, we set up a non-blinded, non-placebo, randomized controlled trial, using a 2 × 3 factorial design. This study design allows for rapid assessment of potential effects of progesterone treatment and dose combinations on breast volume and may serve as a pilot project for a larger study in transgender women at the start of hormone treatment before surgery. This is a mono-center study at the Center of Expertise on Gender Dysphoria of Amsterdam UMC, location VUmc, a national referral center for individuals experiencing gender incongruence.

### Characteristics of participants

Individuals who regularly visit the clinic are eligible to participate, provided that the individual started GAHT after 18 years of age (to eliminate potential effects of puberty suppression), received more than one year of GAHT, underwent vaginoplasty or orchiectomy (to avoid interference from androgen deprivation strategies), has sufficient knowledge of the Dutch language and a BMI 18–30 kg/m2. A potential subject who meets any of the following criteria will be excluded from participation in this study: previous use of progesterone/ progestin (not including cyproterone acetate or oral contraceptives), history of breast augmentation or reduction surgery, active treatment for depression, current use of progesterone/ progestin including cyproterone acetate, severe familial dyslipidemia (e.g. Familial Hypercholesterolemia), serum estradiol concentration > local reference range (200–400 pmol/L) at last visit prior to baseline, contraindications for the use of micronized progesterone, history of epilepsy (because of the oscillating 3D scanner).

### Processes, interventions and comparisons

**Fig. 1:**
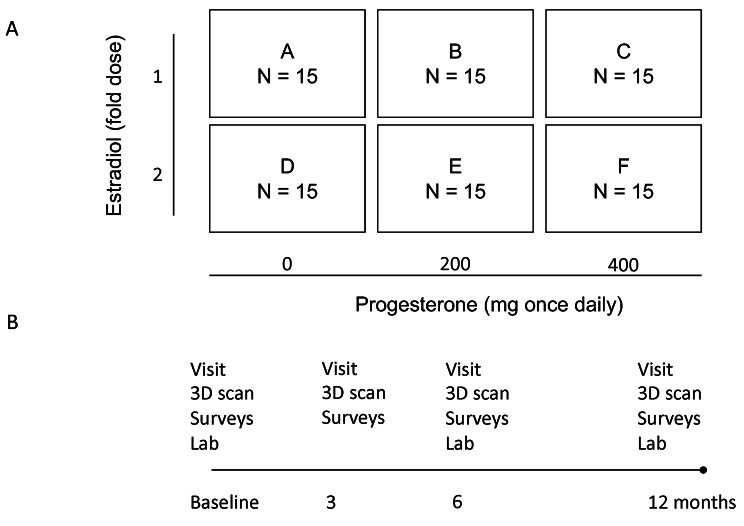
Study set-up. (**A**) Ninety participants will be randomized into six groups of 15 subjects each, receiving either the baseline dose of estradiol (group A), the baseline dose of estradiol and progesterone 200 mg daily (**B**), the baseline dose of estradiol and progesterone 400 mg daily (**C**), twice the baseline dose of estradiol (**D**), twice the baseline dose of estradiol and progesterone 200 mg daily (**E**), or twice the baseline dose of estradiol and progesterone 400 mg daily (**F**). (**B**) The study will include 12 months of follow-up after the start of the intervention. Visits are scheduled at baseline, after 3, 6 and 12 months

The investigational treatment is micronized progesterone (Utrogestan®), in capsules of 100 mg. Progesterone is combined with and analyzed versus pre-defined doses of estradiol: In a 2 × 3 factorial design, the participants are randomized into six groups of 15 participants each, i.e. 90 in total (groups A-F). The intervention takes 12 months. Group A continues to receive the dose of estradiol at baseline and serves as the control group, group B receives the baseline dose of estradiol and micronized progesterone 200 mg every night at bedtime, group C receives the baseline dose of estradiol and micronized progesterone 400 mg every night at bedtime, group D receives twice the baseline dose of estradiol, group E receives the baseline dose of estradiol and micronized progesterone 200 mg every night at bedtime, group F receives twice the baseline dose of estradiol and micronized progesterone 400 mg every night at bedtime (Fig. 1A). In order to maximize adherence, progesterone is prescribed for limited 1–3 month intervals and progesterone concentrations are measured at the 6 and 12 month time points. At the time of submission of the manuscript participant recruiting was ongoing and 86 individuals had been included in the study.

The established feminizing hormone therapy for transgender women after vaginoplasty or orchiectomy consists of estradiol, prescribed as tablets, patches, gel or spray. The baseline dose of estradiol (groups A, B, C) is defined as oral estradiol valerate (Progynova®), or estradiol hemihydrate tablets (generic, Estradiol Sandoz®, Estrofem®, Zumenon®), or transdermal estradiol patches (Systen®, Sandoz®), estradiol gel (Oestrogel®) or spray (Lenzetto®) resulting in serum estradiol concentrations of 200–400 pmol/L, which are the current local reference ranges. Twice the baseline dose of estradiol (groups D, E, F) is defined as doubling of the estradiol dose of the participant at study entry in order to achieve and maintain serum estradiol concentrations of 400–800 pmol/L. These higher concentrations can be considered safe as these are within the reference ranges stated in the international guideline for hormone treatment of gender dysphoria [2].

**Fig. 2:**
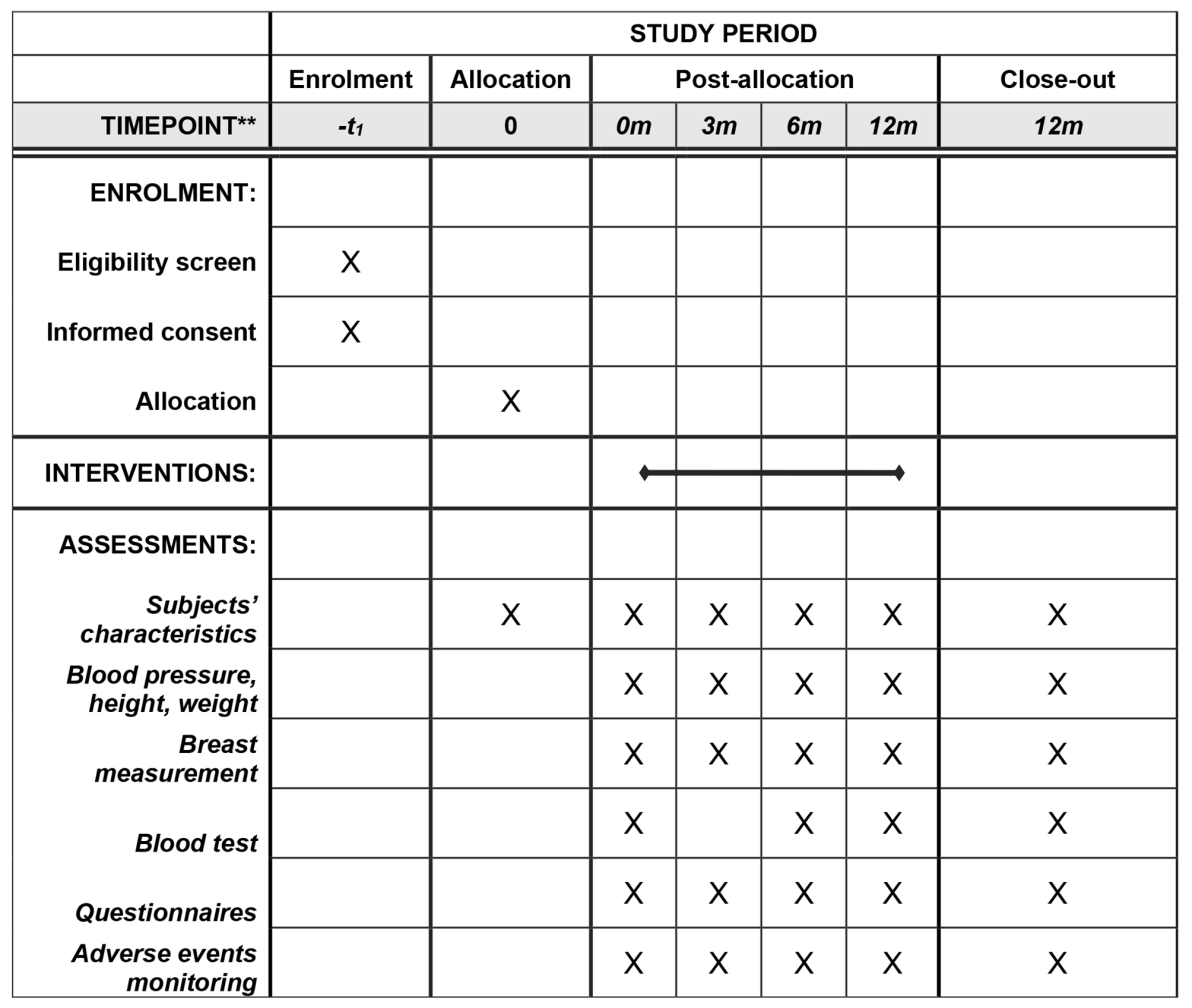
Spirit figure of the trial

Two doses of micronized progesterone are tested in order to investigate a potential dose-response effect. If a dose-response relation is established, this would support a biological effect of the treatment. The duration of the study will be approximately three years in total and will include 12 months of patient follow-up after the start of the intervention (Fig. 1B). Visits are scheduled at baseline, after 3, 6 and 12 months. The study protocol is in line with the Standard Protocol Items: Recommendation for Interventional Trials (SPIRIT) guidelines (Fig. 2).

### Main study parameter/endpoints

The main study end point is breast volume change over the duration of this study. For this purpose, 3D breast images and breast-chest circumference differences are measured (centimeters). 3D imaging of the breasts is carried out using an Artec Leo 3D (Artec 3D, Luxembourg, Luxembourg) scanner in order to determine breast volume. The Artec LEO is a wireless vertical cavity surface-emitting laser scanner (VCSEL) and an improved and more accurate version of the Artec Eva 3D scanner. Previous research has shown that the Artec Eva 3D scanner provides accurate, valid and reliable 3D scans [19, 20]. The Artec LEO can be used with Artec Studio software version 15.1.2.60. All 3D scan files are analyzed in GOM-inspect (GOM, Braunschweig, Germany) and Meshmixer (Autodesk Research, San Francisco, U.S.A.) for breast volume estimation in cubic centimeters (cc). Bra cup sizes will be calculated from breast-chest circumference differences and from the 3D images.

After inclusion, we obtain a complete history (including current or previous illnesses, medication, smoking, alcohol use, family history, previous use of progesterone and cyproterone acetate). At baseline and during follow-up, the following patient characteristics will be recorded: age, height, weight, BMI, blood pressure, alcohol use, smoking, type of estradiol use and breast volume. At the start of the study and after six and twelve months, venous blood samples are obtained and analyzed for determination of concentrations of 17-beta-estradiol, progesterone, luteinizing hormone (LH), follicle-stimulating hormone (FSH), prolactin, sex hormone-binding globulin (SHBG), as well as alkaline phosphatase (APH), gamma-glutamyltransferase (gamma-GT), alanine-aminotransferase (ALAT) and aspartate-aminotransferase (ASAT), low-density lipoprotein (LDL) and HDL cholesterol and triglyceride levels. Estradiol concentrations will be measured using liquid chromatography followed by mass spectrometry. Progesterone, LH, FSH, prolactin and SBHG will be measured using immunoassays.

Satisfaction is assessed at the start of the intervention and during the follow-up visits by three online questionnaires: the Rosenberg self-esteem scale [21], the Breast Satisfaction Rating Scale (BSRS) and a non-validated satisfaction questionnaire about various aspects of the breasts [5]. Satisfaction is measured with regard to several aspects such as symmetry of the breast, the breast volume, and size and shape of the nipple. In addition, adverse and serious adverse effects are recorded. Further, mood and sleep quality will be assessed using the Perceived stress scale, Inventory of Depressive Symptomatology, self-report (IDS-SR) and Pittsburgh Sleep Quality Index (PSQI) questionnaires. Levels of experienced sexual pleasure will be assessed using the Amsterdam Sexual Pleasure Index (ASPI) [22].

### Statistical analysis

All analyses will be performed using the most recent version of STATA. Baseline data will presented as mean with SD for normally distributed data, and median with interquartile ranges for nonnormally distributed data. Data on tobacco use and alcohol use will be presented as percentage of users. The analyses will be blinded with regard to the treatment regimen for the researcher performing the data analyses. Estradiol use will be coded as 0 (normal dosage) or 1 (double dosage), and progesterone will be coded as 0 (no use), 1 (200 mg), or 2 (400 mg). Thereafter, linear mixed model analyses with measurements clustered within participants will be performed, with breast volume as continuous outcome variable and time with set time points at 0, 3, 6, and 12 months as determinants [23]. Estradiol and progesterone use will be analyzed separately and as interaction terms. By performing analyses this way, the separate effects of estradiol and progesterone can be assessed, as well as the effects of the various treatment combinations. Breast size and volume measurements will be recorded in cc. All data are presented as mean change in cc with 95% confidence intervals. Individual-level outliers will be identified with a standardized residual cut-off point of ± 5 [24]. Non-normally distributed data will be log-transformed before mixed-model analyses. The mixed model analysis method can be used and adapted to compensate for missing values and assess potential effect modifying factors. Analyses will be stratified to treatment regimen, age, weight change and serum hormone levels. Age will be analyzed in quartiles and weight change will be assessed in 10 kg groups. Analyses for serum hormone levels will be stratified in quartiles. Satisfaction, mood, sleep, and sexual desire will be recorded as categorical variables. Sexual pleasure will be assessed using a continuous scale of the ASPI score. To analyze the relationship between satisfaction of different aspects of the breasts and breast volume, mixed model analyses will be performed. Treatment regimens, hormone levels and breast volume changes will be assessed as covariates. Side effects and adverse events will be recorded as percentages and analyzed using Fisher’s exact testing.

We considered an average increase of 30% of breast volume – corresponding to one bra cup size increase – as a clinically relevant outcome after one year of study treatment. From previous studies, it is known that the ratio of two consecutive breast volume measurements is 1.08 with a standard deviation of 0.27 [5]. Using a power simulation model (STATA, factorialsim), in which the increase of the estradiol dose is treatment A and addition of progesterone is treatment B in a factorial design of six groups of 15 subjects, at a set alpha of 0.05 the simulated power of A is estimated at 0.95 and the power of B at 0.94.

## Discussion

The goals of this study are to assess effects and side effects of various dose combinations of progesterone and estradiol in a group of transgender individuals, assigned male sex at birth who have undergone orchiectomy or vaginoplasty. The factorial design of the study allows assessing potential synergistic effects of progesterone and estradiol. Estradiol and progesterone have been used for many decades in post-menopausal women and in in vitro fertilization procedures and their safety profiles in these patient groups are well known [17, 25]. Estradiol is the cornerstone of the endocrine treatment of transgender women [2]. The World Professional Association for Transgender Health Standards of Care acknowledge the use of progestins and micronized progesterone in trans women and emphasize a lack of evidence on beneficial effects and potential side-effects [3]. A potential role for progesterone in breast development in transgender women has not been investigated in a randomized controlled experimental set-up. A recent case-controlled study showed that addition of micronized progesterone to feminizing GAHT did not affect Tanner stage in a group of 23 transgender individuals. However, participants in this study used a low dose of progesterone, the follow-up was short, only few individuals had undergone gender-affirming surgery and more than half of the participants also used the progestogen cyproterone acetate. The authors concluded that larger, randomized trials with higher doses of oral progesterone added to feminizing hormone therapy regimens remain necessary [14]. This study protocol includes multiple dose combinations of progesterone and estradiol, enabling studying dose-response relationships and synergistic effects. The study has a 12-month follow-up period and will be carried out in a cohort of subjects who do not use anti-androgens or progestogens at the start of the study. Therefore, we expect that this study will add to our understanding of the effects and side-effects of adding progesterone to GAHT in transgender individuals seeking feminization and guide future efforts for innovation of GAHT.

## Data Availability

The datasets generated and/or analyzed during the current study are not publicly available due to privacy reasons. The corresponding author can be contacted for the availability of data.
